# Combining machine learning and optimization for the operational patient-bed assignment problem

**DOI:** 10.1007/s10729-023-09652-5

**Published:** 2023-11-28

**Authors:** Fabian Schäfer, Manuel Walther, Dominik G. Grimm, Alexander Hübner

**Affiliations:** 1 Technical University of Munich, Campus Straubing for Biotechnology and Sustainability, Supply Chain and Value Management, Straubing, Germany; 2 Catholic University of Eichstätt-Ingolstadt, Supply Chain Management & Operations, Ingolstadt, Germany; 3 Technical University of Munich, Campus Straubing for Biotechnology and Sustainability, Bioinformatics, Straubing, Germany; 4 Weihenstephan-Triesdorf University of Applied Sciences, Bioinformatics, Straubing, Germany; 5 TUM School of Computation, Information and Technology (CIT), Technical University of Munich, Garching, Germany

**Keywords:** Hospital bed management, Patient-room assignment, Stakeholder integration, Emergency forecasting, Emergency patient admissions, Machine learning, Operations research, Operations management

## Abstract

Assigning inpatients to hospital beds impacts patient satisfaction and the workload of nurses and doctors. The assignment is subject to unknown inpatient arrivals, in particular for emergency patients. Hospitals, therefore, need to deal with uncertainty on actual bed requirements and potential shortage situations as bed capacities are limited. This paper develops a model and solution approach for solving the patient bed-assignment problem that is based on a machine learning (ML) approach to forecasting emergency patients. First, it contributes by improving the anticipation of emergency patients using ML approaches, incorporating weather data, time and dates, important local and regional events, as well as current and historical occupancy levels. Drawing on real-life data from a large case hospital, we were able to improve forecasting accuracy for emergency inpatient arrivals. We achieved up to 17% better root mean square error (RMSE) when using ML methods compared to a baseline approach relying on averages for historical arrival rates. We further show that the ML methods outperform time series forecasts. Second, we develop a new hyper-heuristic for solving real-life problem instances based on the pilot method and a specialized greedy look-ahead (GLA) heuristic. When applying the hyper-heuristic in test sets we were able to increase the objective function by up to 5.3% in comparison to the benchmark approach in [40]. A benchmark with a Genetic Algorithm shows also the superiority of the hyper-heuristic. Third, the combination of ML for emergency patient admission forecasting with advanced optimization through the hyper-heuristic allowed us to obtain an improvement of up to 3.3% on a real-life problem.

*Highlights

- Integrating the perspectives and constraints of key stakeholders, including patients, doctors, and nursing staff, ensures holistic patient-bed assignment decisions.
- Employing machine learning techniques to enhance the accuracy of forecasting emergency patient admissions and analyzing features like weather data, time, dates, and local events.
- Demonstrating that machine learning methods outperform conventional time series forecasts, delivering up to a 17% improvement in predicting emergency patient admissions.
- Introducing a novel hyper-heuristic approach to optimize patient bed-assignment scenarios, achieving a remarkable up to 5.3% enhancement in a time series analysis.
- Achieving an overall performance improvement of up to 3.3% in solving real-world patient bed-assignment data sets by synergizing machine learning for emergency patient admission forecasting with advanced optimization via the hyper-heuristic.

**Fig. 1:**
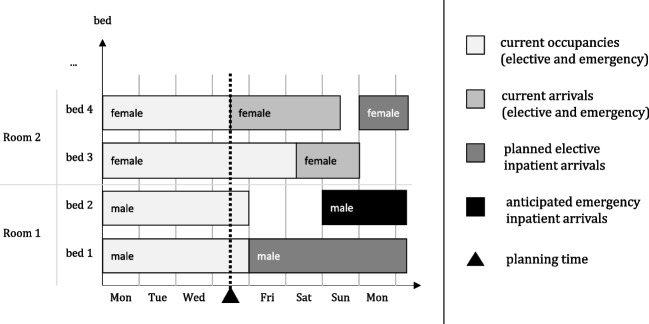
Illustration of the patient bed assignment problem (PBA)

## Introduction

### Context and motivation

Rising life expectancy, higher morbidity, and a changed spectrum of illnesses, but also technical and medical progress, which increasingly makes it possible to treat more and more diseases, are causing demand to rise for hospital treatments and increasing healthcare spending. For example, in Germany, spending rose by 4% in 2018 compared to the previous year and healthcare spending accounted for almost 12% of Gross Domestic Product [43]. Contrary to the increased demand for healthcare services, existing resources were reduced at the hospital level to compensate for the higher spending. From 2000 to 2017, the median number of hospital beds in OECD countries decreased by 13% [37]. In Germany, where a longer data history is available, from 1991 to 2018, there is a reduction of 20% in the number of hospitals, 25% in the number of beds, and a 33% increase in patient numbers [44]. Higher demand needs to be serviced with the scarcity of resources. If this is not to be at the expense of the patients and the quality of medical care, this can only be achieved by optimizing the utilization of available resources.

### Problem description

Hospital beds are one of these scarce resources. To efficiently use hospital beds, they are not any more planned individually by each ward as in the past, but oftentimes for the entire hospital to obtain pooling effects (see e.g., [8, 20, 28]). When arriving at the hospital, patients are directly or after a treatment (e.g., in operating or emergency room) admitted to a ward bed. This operational problem of assigning inpatients to specific rooms and beds is defined as patient-bed assignment problem (PBA). Figure 1 illustrates the PBA and its dependencies.

Two female emergency patients who have just arrived are planned to stay in beds 3 and 4. While bed 1 is theoretically available before bed 3, it is already “reserved” for a male elective patient scheduled to arrive on Friday and stay for several days. Consequently, the female patient planned to occupy bed 3 will have to wait in an overflow area (e.g., hallways, emergency or treatment rooms) until Saturday when bed 3 becomes available for her. Her treatment will begin in the overflow area as a postponement of the emergency patient admission is not medically possible and reasonable. In general, elective patients typically do not accept that a room and bed are not “reserved” for them upon their planned arrival, while emergency patients are more willing to accept having to temporarily stay in dedicated overflow areas due to the unplanned arrival (see [39]). In the example above, it is therefore considered more important that the elective patient arriving on Friday does not have to wait in an overflow area. Hence, it is crucial to determine at which time a specific physical room and bed is to be assigned to an inpatient and whether or not it should be possible to reserve such a bed.

### Challenges in patient-bed-assignment

Efficient real-time planning systems are required in order to guarantee patient satisfaction and trouble-free process flow (e.g., avoid waiting times until inpatient admission as well as blocking emergency departments). The complexity of the PBA results from different stakeholder needs, frequent changes in lengths of stay (LOS) and estimating the number of beds required. First of all, the PBA needs to bring together the interests of patients, doctors, and nurses. To facilitate doing rounds and patient visits, walking distances for doctors should be minimized. This can be achieved by grouping similar patients, i.e., patients associated with a specific department, into rooms. In contrast to doctors, nurses tend to a broader range of patients. However, they are typically dedicated to a specific ward, working in well-coordinated teams, and therefore cannot easily be transferred to other wards. Thus, balancing the workload between wards is a key objective for nurses when assigning patients to beds. Hence, the PBA affects patient satisfaction (e.g., immediately available bed, suitable room with adequate roommates), the workload of nurses (e.g., a mix of work-intensive and easy-to-handle patients), and workload of doctors (e.g., own patients located in proximity). These may comprise some trade-offs. For example, focusing only on patient satisfaction by putting optimal roommates together (i.e., patients of similar age or with similar illnesses) may be in conflict with the nurse workload. Second, deviations from expected medical conditions and treatment plans are normal, for example, if patients remain in intensive care units (ICU), LOS changes happen (e.g., earlier or later discharge, unforeseen surgical complications, newly detected medical conditions), elective inpatients do not arrive or different medical infrastructure becomes necessary. Whenever one of the events takes place, the PBA plan needs to be updated. This can easily affect 50% of all beds per day if, for example, 30% of the inpatients are discharged and admitted per day and 20% are affected by LOS updates. Finally, a further complication arouses from the inherent uncertainty of bed needs for emergency inpatients which may account for up to 90% of all inpatients. Appropriately predicting which kind of emergency patients and how many from each hospital department are likely to arrive is a fundamental input to the PBA. Several external effects like seasons, weather, or local events and different drivers for each discipline like snowy weather for trauma surgery may drive the volume of emergency patients.

### Contribution

The main body of related literature considers elective patients only (see e.g., [6, 16, 32]). In these applications, assignment is done for a known set of patients to a given set of empty beds. Some extensions deal with dynamically arriving elective patients (see e.g., [13]) and uncertainty in LOS (see e.g., [36]). The present paper builds upon the extended model introduced by [40] that copes with patient-, nurse- and doctor-specific criteria, and accounts for dynamically arriving elective and emergency patients as well as dynamic changes of LOS. They apply an average emergency inpatient arrival rate. However, simply predicting emergency patients based on historical averages will be suboptimal, as it seems highly probable that the actual number of emergency admissions is dependent on a plethora of internal and external factors in each medical specialty. We extend this approach by proposing a ML approach to anticipate future emergency inpatient arrivals. The analysis is based on a comprehensive empirical data collection (e.g., patient data, weather, regional events). This allows us to investigate factors that can predict emergency admissions for each medical specialty and analyze the impact of improving the forecasts on bed planning employing numerical studies with actual data from a maximum-care hospital. ML will continue to revolutionizing healthcare management due to the exponential increase of data and computing power (see, e.g., [3]). Predictive analytics will be entering the space of operational management in hospitals and improve decision making (see, e.g., [27]). We develop insights in the use of ML on emergency patient admission forecasting. We further contribute with an advanced solution approach by tailoring a hyper-heuristic framework to the PBA. We combine the forecasts obtained with ML with a hyper-heuristic framework for solving the PBA efficiently for large problem instances within a real-life application.

The remainder of this paper is organized as follows. We review related literature in Section 2. The underlying mathematical model of our decision problem and advanced optimization approach is summarized in Section 3. Section 4 provides several numerical examples based on actual hospital data. Finally, Section 5 presents a summary of the main results and outlines potential avenues for further research.

## Related literature and research gap

We contribute to the PBA literature and the related forecasting of emergency patients. The literature can be structured along (i) static and (ii) dynamic models for PBA and (iii) forecasting models for bed requirements.

### (i) Static models for PBA

The static PBA was first introduced by [16]. They consider a situation in which a hospital is initially empty and all future patient arrivals within a given time horizon are deterministically known as well as their respective parameters, e.g., actual LOS, gender, department adherence, and individual infrastructural needs. Patients are assigned to rooms such that an overall objective function based on violating patient-specific requirements is minimized. Capacity is assumed to be sufficient to accommodate all inpatients. As such, it does not allow for shortage situations. Furthermore, they do not distinguish between emergency and elective patients. Several authors like [6, 9, 13, 17, 23, 32, 47] have since built on the model of [16] by providing alternative solution approaches like matheuristics such as the Genetic Algorithm (GA). For details, we refer to Table 1. To summarize, the static models assign elective patients to beds. However, whenever a patient is admitted to or discharged from wards, patients are reassigned from the overflow, no-shows of elective patients occur, sudden changes in medical infrastructural requirements, an unexpected need for medical isolation or changes in the LOS become necessary, the static plan is no longer valid. The PBA need to be updated. Therefore, the static models provide only a starting point for solving the PBA.

**Table 1:** Overview of decision models related to patient bed assignment

		Forecast	PBA model					Analyses	
Contribution	Solution approach	Emergency $^{1}$	Problem $^{2}$	Patients $^{3}$	Overflow $^{4}$	Uncertainty $^{5}$	Stakeholders$^{6}$	Rolling horizon $^{7}$	Data sets used $^{8}$
Demeester et al. [16]	Hybrid tabu search	–	S	El	–	–	P	–	S
Bilgin et al. [9]	Hyper-heuristic	–	S	El	–	–	P	–	S
Kifah and Abdullah [32]	Great deluge	–	S	El	–	–	P	–	S
Turhan and Bilgen [47]	Fix-and-optimize heuristic	–	S	El	–	–	P	–	S
Guido et al. [23]	Matheuristic	–	S	El	–	–	P	–	S
Bastos et al. [6]	Exact approach	–	S	El	–	–	P	–	S
Dorgham et al. [17]	GA	–	S	El	–	–	P	–	S
Ceschia and Schaerf [13]	LNS	–	S,D	El	–	$\checkmark$	P	–	S
Ceschia and Schaerf [14]	LNS	–	S,D	El,Em	–	$\checkmark$	P	–	S
Ceschia and Schaerf [15]	LNS	–	S,D	El,Em	–	$\checkmark$	P	–	S
Lusby et al. [36]	Adaptive LNS	–	D	El,Em	–	$\checkmark$	P	–	S
Vancroonenburg et al. [48]	Specialized heuristic	–	S,D	El,Em	–	$\checkmark$	P	–	S
Schäfer et al. [40]	Greedy heuristic	–	S,D	El,Em	$\checkmark$	$\checkmark$	P,N,D	$\checkmark$	S,R
This paper	Hyper-heuristic, GA	TSF, ML	S,D	El,Em	$\checkmark$	$\checkmark$	P,N,D	$\checkmark$	R

$^{1}$ Emergency inpatient arrival forecast (analysis of effects beyond using simple historical averages)

$^{2}$ S := static version of the PBA (every future arrival known, no prior occupancy);

D := dynamic version of the PBA (prior occupancy considered, arrivals known within planning horizon)

$^{3}$ El := elective patients; Em := emergency patients

$^{4}$ Situations in which not enough beds are available and patients have to spend time in an overflow buffer until a bed becomes available

$^{5}$ Uncertainty regarding patient LOS and future admission dates

$^{6}$ Objectives and constraints considered for patients (P), nurses (N), and doctors (D)

$^{7}$ Planning with rolling horizon of the PBA (analysis of effect that continuous plan updates have over the course of several weeks)

$^{8}$ S := simulated problem instances; R := real hospital data

### (ii) Dynamic models for patient bed assignments

Ceschia and Schaerf [13] are the first to provide an approach for adapting the PBA model to the dynamic case. To this end, they include the arrival date of the patient. The number of days an arrival is known in advance can vary for elective patients and can be considered to equal zero for emergency arrivals. In addition, they consider pre-occupancies, i.e., patients who are already in the hospital at the planning date. Ceschia and Schaerf [13] apply a large neighborhood search (LNS) and provide an approach to investigating the uncertainty regarding the discharge date of a patient. To assess the impact of different LOS they solve the PBA several times using different values for the discharge dates of all patients in the system. Ceschia and Schaerf [14] and Ceschia and Schaerf [15] include uncertainty by factoring in flexible horizons and patient delays while also adding operating room constraints. Based on the work of [14, 36] apply an adaptive LNS. Vancroonenburg et al. [48] introduce a model that is designed to only assign those patients to a new room who have just arrived and physically require a bed. In addition, they suggest a second model in which they also assign patients to beds who are registered in the system but have not yet arrived. Finally, [40] show that the PBA is a multi-objective problem that needs to ensure patient satisfaction and optimize workloads for nurses and doctors at the same time. The goals and constraints of patients, nurses, and doctors for PBA need to be considered simultaneously. Their model further incorporates patient-patient dependencies concerning rooms and wards, distinguishes between emergency and elective patients, and incorporates their respective needs. The number of emergency patients is estimated with historical averages. Patients may be allocated to overflow buffers to reserve beds for other patients or compensate for temporarily unavailable beds. They apply a greedy look-ahead (GLA) heuristic for dynamically arriving patients with uncertainty in emergency inpatient arrivals and LOS. To summarize, the dynamic models extend the static models by considering dynamically arriving elective and emergency patients. It is shown that emergency patients have a strong influence on bed assignments. This calls for appropriately predicting which kind of emergency patients and how many are likely to arrive. Only [40] provide an approach to estimate emergency patients based on historical distributions, however, without further investigating the impact of better estimates on PBA. As such, we extend the review by analyzing related literature with emergency forecasts.

### (iii) Literature related to estimating emergency patients and bed occupancy

General reviews on forecasting emergency arrivals by [24, 50], e.g., on outpatient arrivals, day clinic walk-ins, or emergency calls, summarize different approaches and goals. With respect to PBA, [12] as well as [1] concern themselves with the problem of forecasting emergency arrivals at a hospital. Both use hospital data and use an autoregressive moving average approach. Schiele et al. [41] provide a model to anticipate resulting bed occupancy levels based on a given master surgery schedule. They consider different patient types and paths and make use of a neural network approach to improve their prediction quality.

### Summary and research gap

The PBA has gained more and more attention within the past decade. Key challenges dealt with in most contributions to this area of research can be seen in the computational complexity as well as the underlying uncertainty and volatility of most parameters involved. Table 1 summarizes the most recent contributions and highlights a set of key aspects related to the challenges mentioned above.

In general, we can constitute three major research gaps:

- **(1) Anticipating emergency arrivals in PBA:** The current PBA models apply, if emergency patients are considered, only average values from the past. However, it seems highly probable that the actual number of emergency admissions is dependent on a plethora of internal and external factors to the hospital. Several external effects may drive the volume of emergency patients, e.g., seasons, weekdays, and local events like county fairs or sports events. There may be different drivers for each specialty like snowy weather for trauma surgery, and the availability of family doctors for internal medicine.
- **(2) Advanced solution approach with uncertainty and dynamic online planning:** For such real-life applications, it is important to have a solution approach that is proven to work in this dynamic environment with frequent and short-term events that cause plan adaptions. That means at each point in time that an inpatient gets admitted or discharged or when any other change in the system merits moving patients from an overflow area to a regular bed. In addition, the underlying uncertainty typically requires several adaptations of future PBAs during any given day.
- **(3) Application to real-life settings:** The vast majority of models is applied to simulated data sets. Furthermore, actual hospital situations like shortage of beds (and hence overflow situations) and multiple stakeholders and their trade-offs are scarcely integrated.

This paper addresses these open areas by (i) applying time series forecasts (TSF) and ML approaches for the forecasting of emergency patient admissions and assessing the impact of better forecasts on PBA, (ii) developing an advanced heuristic tailored to the dynamic online planning problem at hand, and combining these for (iii) deploying it in real-life scenarios. The case study is conducted with a large German maximum-care hospital. Concerning (i), a broader investigation with main features by medical disciplines, including metadata and testing the impact of better emergency forecasts on PBA becomes necessary. This will allow the prediction of emergency admissions more accurately compared to solely drawing on historical distributions of patient arrivals. Such an approach is promising as it relies on publicly available data and as such is possible to be incorporated in the planning systems of hospitals. We then investigate how and to which extent sophisticated forecasts can help to advance the planning quality of PBA. Concerning (ii), we will improve the solution approach of [40]. This is the most related model to this paper. Their focus was on the introduction of a comprehensive modeling approach and the introduction of the multi-objective problem. We will apply the identical decision model as it incorporates the various stakeholder requirements found in practice. However, the solution approach of [40] is limited to a GLA heuristic. We will further develop this to a hyper-heuristic that incorporates elements of the pilot method introduced by [18]. Finally, in (iii) we reconcile the insights gained in (i) and (ii) and apply them to a real-world case study.

## Forecasting, decision model and solution approach

This section details first the approach for forecasting emergency patients using TSF and ML methods. This serves as input for the decision model that is outlined next, before the introduction of the developed hyper-heuristic.

### Emergency patient admissions related feature importance and their prediction

It is expected that external influences like weather, seasons, or events have an impact on the emergency volume. Hence, to estimate emergency patient admissions metadata that is suspected of having an impact on patient volume in the emergency department has to be gathered. First, to get a deeper understanding of the distinct features and how they influence the emergency arrivals the *(1) importance of features* are computed. The results allow for deriving managerial insights. For example, which features are particularly important or whether there are differences between individual specialties. This step is helpful for understanding and allows us to easily derive thumb rules for practice applications. In contrast, ML methods often act as a black box. Second, different forecasting methods are suitable for the concrete *(2) prediction of emergency patient admissions*.

#### Importance of features

In the first step, to avoid multicollinearity issues (see e.g., [25]), the Pearson correlation coefficients (PCC) of each potential pairing of features are determined. Positive and negative high correlating pairs are detected and only one variable is used for each of these pairings for the further procedure. The remaining features have to be tested to determine their explanatory power regarding the number of patient arrivals on a given day. This is important for two reasons. First, simply looking at the direct correlation between a given feature and the number of emergency arrivals in the test data can be misleading as this overlooks any potential effects that certain properties only have in combination [25]. Second, ML algorithms tend to be overfitted when the number of features used is significantly higher than optimal (see for example [33]). To this end, we make use of the “Boruta” package developed by [34]. It consists of a feature selection algorithm based on the “random forest” classification method [11]. It aims to rank a set of features according to their respective predictive power regarding a specific classification or regression variable, e.g., the number of emergency patient arrivals per day. This ranking is performed according to the individual “importance” of each feature, which is based on the average and standard deviation of the loss of accuracy of classification caused by the random permutation of attribute values between objects. A key idea here is to introduce so-called “shadow variables”, i.e., additional random variables, which are then included in the set of existing features. By adding randomness to the data set and collecting results from the ensemble of randomized samples, it is possible to reduce the misleading impact of random fluctuations and correlations.

#### Prediction of emergency patient admissions

Estimating the number of emergency patient admissions is inherently a regression problem. Disregarding the metadata, the problem can be simplified and solved with *(1) time series forecasting* (TSF) techniques. To incorporate metadata, *(2) regression-based methods* and *(3) a multilayer artificial neural network* (ANN) is presented. In contrast to regression-based methods, ANN takes into account nonlinear dependencies. Note that these three different approaches also serve as benchmarks in our numerical tests.

##### (1) Time series forecasting

TSFs estimate future values based on previously observed time series values. Advanced TSF methods take into account the influences of level, trend, and seasonality in the time series. The Holt-Winters method [51], also known as triple exponential smoothing, and Seasonal Autoregressive and Integrated Moving Average (SARIMA) models (see [10]), take into account all three aforementioned components. The Holt-Winters method is an exponentially weighted moving average for determining the level, trend, and seasonal components of a time series. The smoothing parameters are set to minimize the squared error in the one-step-ahead prediction. The SARIMA model identification and parameter determination usually take place through a systematic process of testing. Neither method outperforms the other. Their performance depends on the problem and is therefore often compared in the literature.

##### (2) Regression-based methods

Since it is not known which underlying effects the features exhibit, various regression models should be considered. Ridge regression (RR) uses $l_2$-regularization [26], whereas LASSO (LR) uses $l_1$-regularization [46]. $l_2$-regularization accounts for correlations between the input features, while $l_1$-regularization favors sparse solutions. Elastic Net (EN) is a regression-based method that combines $l_1$ and $l_2$ regularization [53]. Another class of regression models is Group-LASSO (GL), which allows individual features to be combined into groups [52]. All features of a group are penalized together, leading to whole groups being considered or neglected.

##### (3) Artificial neural network

ANN is used to account for non-linear dependencies [22, 35]. A neuron is the building block of each ANN which comprises two mathematical operations. First, it computes the weighted average of its input values plus a bias. The resulting sum is passed through a non-linear activation function. Neurons can be combined into larger structures that build an additional layer (called hidden layer). A hidden layer serves as a connection between the first (input) and last (output) layers. Several typologies for ANN can be determined by varying the number of hidden layers. Each layer is fed by the outputs of the previous layer. The formulated ANN is optimized by a specific solver according to a specific loss function.

### Decision model

#### General idea

The underlying problem of the PBA could be represented as a stochastic dynamic program. The dynamic setting of the problem arises from multiple events such as arrivals, discharges, and no-shows of patients as well as changes in LOS. Here, each event represents a stage and the total number of inpatients constitutes the state space in each stage. To illustrate, when assuming the case of a large hospital with about 800 beds occupied on average, an average of over 500 events per day of these beds, and a planning horizon of 28 days, this would result in more than 14,000 stages and a total state space of more than 11 million entries. The stochastic volatility arises from the fact that the total number and type of inpatients cannot be predetermined and are further subject to uncontrollable external influences (such as weather, patient recovery, treatment complications, etc.). In light of the stochastics and high number of dynamically emerging events, it is almost impossible to optimally solve such a dynamic problem setting for actual hospital applications, meaning that a heuristic approach is required to provide efficient and effective decision support in real-life settings. We approximate the dynamic problem as [40] by solving a static model that is updated at each possible event. Ceschia and Schaerf [13] propose a similar approach to test the performance of their static model in a dynamic setting. When solving the model, it allocates beds for patients (new inpatients and patients from the overflow buffer), assigns patients to overflow, and reserves beds for patients (currently in overflow and future patient arrivals). As such, we subsequently solve single stages while considering future arrivals and discharges that are both already known and estimated. The model takes all the relevant information currently available into account for each of these individual stages.

#### Model overview

The decision model is based on [40]. We model the identical problem, but introduce different subsets to obtain a much more compact formulation. Table 2 summarizes the notation.

**Table 2:** Notation

Sets	
*B*	Set of beds, $B=\{1,2,...,b,...,|B|\}$
$B_{p}$	Subset of beds *B* which is available for patient *p*
$B_r$ ($B_w$)	Subset of beds *B* which are located in room *r* (in ward *w*)
*D*	Set of medical departments, $D=\{1,2,...,d,...,|D|\}$
*P*	Set of inpatients, $P=\{1,2,...,p,...,|P|\}$
$P_{b,t}$	Subset of inpatients *P* who fit in bed *b* on day *t*
*R*	Set of rooms, $R=\{1,2,...,r,...,|R|\}$
*T*	Set of days within the planning horizon, $T=\{1,2,...,t,...,|T|\}$
*W*	Set of wards, $W=\{1,2,...,w,...,|W|\}$
Parameters	
$\alpha , \beta , \gamma , \delta$	Weights for basic and extended patient-, doctor- and nurse-related utilities, respectively
$\mathrm {\Xi }_{p}$	Weight for patients *p* (e.g., elective vs. emergency patient)
$a_p$	Age of patient *p*
$A^{\textrm{max}}_{r,t}$ $\left( A^{\textrm{min}}_{r,t}\right)$	Maximum (minimum) age of all patients already occupying room *r* on day *t*
$C_{w,t}$	Spare care capacity for caring for further patients on ward *w* on day *t*
$c_p$	Care level required to accommodate patient *p*
$D_{r,t}$	1 if all prior occupants of room *r* on day *t* belong to same medical department; 0 otherwise
$d_p$	Medical department of patient *p* with $d_p \in D$
$F_{r,t}$	1 if room *r* is initially empty on day *t*; 0 otherwise
$g_p$	$-1$ if patient *p* is male; 1 if patient *p* is female
*M*	Large integer value “Big M”
$\textrm{OF}_{p}$	Utility parameter of patient *p* depending on the time patient *p* has already spent in overflow
$Q_{t}$	Time-dependent relevance value that arrivals/discharges will take place as anticipated/planned on day *t*
Decision variable	
$x_{b,p}$	1 if patient *p* is assigned to bed *b*; 0 otherwise
Auxiliary variables	
$a^{\textrm{max}}_{r,t}$ $\left( a^{\textrm{min}}_{r,t}\right)$	Maximum (minimum) age of all patients assigned to room *r* on day *t*
$o_{w,t}^+$	Amount the total care capacity on ward *w* on day *t* is exceeded
$y_{r,t}$ ($z_{r,t}$)	1 if all patients assigned to an empty (partially occupied) room *r* on day *t* are from the same medical department; 0 otherwise

The objective function of Eq. (1) maximizes the total utility *U* and consists of four terms that represent basic patient-specific, extended patient-specific, doctor-specific, and finally nurse-specific objectives. The four partial utilities are combined by using the weighted sum method with the factors $\alpha$, $\beta$, $\gamma$, and $\delta$. All four utility values depend on the binary assignment variable $x_{b,p}$ that represents whether a patient *p* is allocated to bed *b*. The model is formulated as follows:

1 $$\begin{aligned} \textrm{maximize}~U= & {} \alpha \cdot \hspace{1mm} \sum \limits _{p \in P} \sum \limits _{b \in B_p} (\textrm{OF}_p + \Xi _p \cdot \sum \limits _{t \in T: P_{b,t} \ne \emptyset } Q_t) \cdot x_{b,p}\nonumber \\{} & {} - \beta \hspace{1mm} \sum \limits _{r \in R} \sum \limits _{t \in T} (a^{\textrm{max}}_{r,t} - a^{\textrm{min}}_{r,t}) \nonumber \\{} & {} + \gamma \cdot \hspace{1mm} \left[ \sum \limits _{r \in R} \sum \limits _{t \in T} F_{r,t} \cdot y_{r,t} + \sum \limits _{r \in R} \sum \limits _{t \in T} (1-F_{r,t}) \cdot z_{r,t}\right] \nonumber \\{} & {} - \delta \cdot \hspace{1mm} (\sum \limits _{w \in W} \sum \limits _{t \in T} o_{w,t}^+) \end{aligned}$$

subject to

2 $$\begin{aligned}{} & {} \!\! \sum \limits _{b \in B_{p}} x_{b,p} \le 1 \qquad \qquad \qquad \qquad \qquad \qquad \qquad \qquad \qquad \qquad \qquad \qquad \;\; \forall p \in P \end{aligned}$$

3 $$\begin{aligned}{} & {} \!\! \sum \limits _{p \in P_{b,t}} x_{b,p} \le 1 \qquad \qquad \qquad \qquad \qquad \qquad \qquad \qquad \;\; \forall b \in B_r; r \in R; t \in T \end{aligned}$$

4 $$\begin{aligned}{} & {} \!\! g_p \!\cdot \! x_{b,p} \!-\! g_{p'} \!\cdot \! x_{b',p'} \!\ge \! -\!1 \quad \forall b,b' \!\in \! B_r; p \!\in \! P_{b,t}; p'\! \in \! P_{b',t}; r \!\in \! R; t \in T \end{aligned}$$

5 $$\begin{aligned}{} & {} \!\! a^{\textrm{max}}_{r,t} \ge A^{\textrm{max}}_{r,t} \qquad \qquad \qquad \qquad \qquad \qquad \qquad \qquad \qquad \qquad \quad \;\; \forall r \in R; t \in T\end{aligned}$$

6 $$\begin{aligned}{} & {} \!\! a^{\textrm{max}}_{r,t} \ge a_p \cdot x_{b,p} \qquad \qquad \qquad \qquad \qquad \; \, \forall b \in B_r; p \in P_{b,t}; r \in R; t \in T\end{aligned}$$

7 $$\begin{aligned}{} & {} \!\! a^{\textrm{min}}_{r,t} \le A^{\textrm{min}}_{r,t} \qquad \qquad \qquad \qquad \qquad \qquad \qquad \qquad \qquad \qquad \qquad \forall r \in R; t \in T\end{aligned}$$

8 $$\begin{aligned}{} & {} \!\! a^{\textrm{min}}_{r,t} \le \sum \limits _{b \in B_r} \sum \limits _{p \in P_{b,t}} A^{\textrm{min}}_{r,t} \cdot x_{b,p} \qquad \qquad \qquad \qquad \qquad \qquad \quad \; \forall r \in R; t \in T\end{aligned}$$

9 $$\begin{aligned}{} & {} \!\! a^{\textrm{min}}_{r,t}\! \le \! a_p \cdot x_{b,p} \!+\! A^{\textrm{min}}_{r,t} \cdot (1\!-\!x_{b,p}) \quad \quad \quad \, \forall p \!\in \!P_{b,t}; r \!\in \! R; b \!\in \!B_r; t \!\in \! T\end{aligned}$$

10 $$\begin{aligned}{} & {} \!\! d_p \!\cdot \! x_{b,p} \!-\! d_{p'} \!\cdot \! x_{b',p'} \!\ge \! \!-\!\textrm{M} \cdot (1\!-\!y_{r,t}) \nonumber \\{} & {} \qquad \qquad \qquad \qquad \qquad \qquad \quad \forall b,b' \!\in \! B_r; p \!\in \! P_{b,t}; p' \!\in \! P_{b',t}; r \in R; t \!\in \! T\end{aligned}$$

11 $$\begin{aligned}{} & {} \!\! \sum \limits _{b \in B_r} \sum \limits _{p \in P_{b,t}} x_{b,p} \ge y_{r,t} \qquad \qquad \qquad \qquad \qquad \qquad \qquad \qquad \quad \, \forall r \in R; t \in T \end{aligned}$$

12 $$\begin{aligned}{} & {} \!\! d_p \cdot x_{b,p} - D_{r,t} \le \textrm{M} \cdot (1-z_{r,t}) \qquad \forall b \in B_r; p \in P_{b,t}; r \in R; t \in T \end{aligned}$$

13 $$\begin{aligned}{} & {} \!\! D_{r,t} - d_p \cdot x_{b,p} \le \textrm{M} \cdot (1-z_{r,t}) \qquad \forall b \in B_r; p \in P_{b,t}; r \in R; t \in T \end{aligned}$$

14 $$\begin{aligned}{} & {} \!\! \sum \limits _{b \in B_r} \sum \limits _{p \in P_{b,t}} x_{b,p} \ge z_{r,t} \qquad \qquad \qquad \qquad \qquad \qquad \qquad \quad \qquad \, \forall r \in R; t \in T \end{aligned}$$

15 $$\begin{aligned}{} & {} \!\! \sum \limits _{b \in B_w} \sum \limits _{p \in P_{b,t}} c_p \cdot x_{b,p} \le C_{w,t} + o_{w,t}^+ \qquad \qquad \qquad \qquad \quad \;\;\, \forall t \in T; w \in W \end{aligned}$$

16 $$\begin{aligned}{} & {} \!\! o_{w,t}^+ \ge 0 \qquad \qquad \qquad \qquad \qquad \qquad \qquad \qquad \qquad \qquad \qquad \quad \; \forall t \in T; w \in W \end{aligned}$$

17 $$\begin{aligned}{} & {} \!\! x_{b,p}, y_{r,t}, z_{r,t} \!\in \! \left\{ 0,1\right\} ; a^{\textrm{max}}_{r,t}, a^{\textrm{min}}_{r,t} \!\in \! \mathbb {N}_0 \quad \, \forall b \!\in \! B; p \!\in \! P_{b,t}; r \!\in \! R; t \!\in \! T \end{aligned}$$

The first term of the objective function Eq. (1) summarizes the basic patient-specific utility of assigning patient $p \in P$ to bed $b \in B_p$. Every assignment of a patient *p* to a bed *b*, i.e., $x_{b,p}=1$ generates a utility that accounts for the days that patient *p* is presumed to spend in bed *b* within the planning horizon *T*. The utility depends on the time the patient *p* already spent in the overflow ($\textrm{OF}_p$) in the past, a patient type-specific factor ($\Xi _p$), bed availability ($P_{b,t}$), and a relevance value ($Q_t$). $\textrm{OF}_p$ allows patients already waiting to be prioritized over patients who have just arrived. $\Xi _p$ is a factor that makes it possible to prioritize between patient types, i.e., elective patients, emergency patients, or patients with special requirements. $P_{b,t}$ indicates the patients *p* who can be assigned to bed *b* on day *t*. This includes the availability of a bed (i.e., not pre-occupied or reserved), avoiding gender mixing (with respect to current occupants), infrastructural constraints as well as medical isolation constraints (with respect to current occupants). $Q_t$ reflects the time-dependent relevance of a bed assignment for patients on day *t* as anticipated/planned, where $Q_t$ decreases with increasing *t*. It gives a higher value to patients who arrive earlier than those who come later in the planning horizon. Due to uncertainties it is quite reasonable that a patient who is planned to arrive far in the future will be reassigned to another bed during later planning periods, which may even lead to a higher overall utility value for that patient. The second term of the objective function represents the goal to minimize the differences between patients within rooms since it is desirable to combine similar patients. The calculation $a^{\textrm{max}}_{r,t} - a^{\textrm{min}}_{r,t}$ denotes the difference between the maximum value and the minimum value of patients in room *r* on day *t*. We use age difference as an indicator for the compatibility between patients (see also [39]). The third term rewards assigning patients from the same department to the same rooms for facilitating medical rounds and reducing walking distances for doctors. Therefore, $F_{r,t}$ is needed, which is 1 if room *r* is empty on day *t*, and 0 otherwise. The fourth term is used to balance the workload for nursing staff. The number of “care units” for each patient, represented by $c_p$, reflects the individual effort and resources required, while the overall “care capacity” per ward *w* and day *t* is constrained by staffing schedules. Parameter $C_{w,t}$ represents the available capacity of a ward *w* on day *t* for new patients, while the auxiliary variable $o_{w,t}^+$ indicates the amount by which the capacity of ward *w* on day *t* is exceeded, penalizing the exceeding of the predefined care capacity.

**Table 3:** Expanded notation for the pilot method

$a_0$	Most promising element $u(a_0) \ge u(a)$ $\forall a \in A$
*A*	Set of all possible choices *a*, so-called pilots
*H*	Subheuristic applied to assign remaining pilots $a \in A \setminus S_a$ (e.g., greedy heuristic)
*N*	Number of partial solutions considered at each iteration
$S_a$	Partial solution $S_a = a \cup X$
*u*(*a*)	Predetermined utility function $u:A \rightarrow \mathbb {R}$
*X*	Master solution, iteratively created by adding the most promising element of an iteration $X = X \cup a_0$

Equations (2) prevent double booking, i.e., a patient can only be allocated to a maximum of one bed. Equations (3) prevent overbooking, i.e., no two patients can be allocated to the same bed on the same day. In addition, Eqs. 4 ensure that there are no mixed male and female rooms on any given day *t*. A similar approach might ensure that medical isolation requirements are respected. Both auxiliary variables $a^{\textrm{max}}_{r,t}$ and $a^{\textrm{min}}_{r,t}$ are dependent on $x_{b,p}$ as well as on the patients already occupying beds. $A^{\textrm{max}}_{r,t}$ ($A^{\textrm{min}}_{r,t}$) is set to the current maximum (minimum) age of all patients already occupying room *r* on day *t*. If room *r* is empty on day *t*, $A^{\textrm{min}}_{r,t}$ is set to a large integer value that represents the maximum possible age (e.g., 120), and $A^{\textrm{max}}_{r,t}$ is set to 0. Equations (5) and (6) ensure that the auxiliary variable $a^{\textrm{max}}_{r,t}$ reflects the maximum age of prior occupants and newly allocated patients in a room *r* on day *t*. Likewise, Eqs. (7) to (9) ensure the same for $a^{\textrm{min}}_{r,t}$ while also making sure that $a^{\textrm{min}}_{r,t}$ equals $a^{\textrm{max}}_{r,t}$ in the event that room *r* is only occupied by one person or completely empty on day *t*. The two auxiliary variables $y_{r,t}$ and $z_{r,t}$ are applied as follows:

- Empty rooms: $y_{r,t}$ is set to 1 if all patients assigned to an empty room *r* on day *t* are from the same medical department, which is achieved by Eqs. (10) and (11). Here, $d_p$ is an integer value that depicts the medical department of patient *p* and *M* represents a large integer value (“big M”), i.e. the maximum indicator number of the departments.
- Occupied rooms: $z_{r,t}$ is set to 1 only if all patients assigned to room *r* are already from the same department. This is achieved by Eqs. (12) to (14). Here, $D_{r,t}$ is set to 1 if all prior occupants of room *r* on day *t* belong to the same medical department, and 0 otherwise.

Equations (15) and (16) link $x_{b,p}$ to $o_{w,t}^+$.

### Hyper-heuristic

This subsection develops the solution approach. Bed managers require a time-efficient system in everyday work that provides real-time decision support for each new event. An optimal solution approach is impracticable with respect to the combinatorial complexity of the PBA. Other approaches in the literature (see for example [13, 16]) also had to resort to using heuristic approaches for the same reasons. Schäfer et al. [40] propose a GLA heuristic that derived from the idea of [5]. It is able to solve the problem time efficiently, but is vulnerable to ending up in a non-optimal solution. To circumvent these types of situations, we develop a hyper-heuristic framework based on the “pilot method” of [18]. It supports greedy algorithms in avoiding local optimum traps. Duin and Voß [18] and Voß et al. [49] show that the pilot method is suitable for solving highly combinatorial problems (like the PBA) and that it performs competitively compared to well-known metaheuristics. By only looking forward, the method iteratively weights all options before choosing the most promising. Further notation is delineated in Table 3.

#### General algorithm

An initial empty master solution $X = \emptyset$ is iteratively supplemented by an element $a \in A$, whereas *A* represents the set of all possible choices, so-called pilots. Based on the master solution *X*, several partial solutions *N* are generated by randomly drawing a pilot ($S_a = a \cup X$). Each partial solution is completed by the remaining pilots $a \in A \setminus S_a$ by applying a subheuristic *H*. Each solution can be evaluated using a predetermined utility function $u:A \rightarrow \mathbb {R}$. Let $a_0$ be the most promising element $u(a_0) \ge u(a)$ $\forall a \in A$. The pilot $a_0$ gets included in the master solution $X = X \cup a_0$ and excluded from the remaining choices $A = A \setminus a_0$. Then the algorithm loops to create the next partial solution $S_a = a \cup X$ until a stop criterion is met (e.g., set of pilots is empty $A = \emptyset$, limitation of iterations). In our case, the utility is the total utility of the objective function of Eq. (1), i.e., $u(a)=U$.

To speed up the computations we limit the solution space by only considering the set of relevant beds $\overline{B}$ and patients $\overline{P}$. The relevant beds considered include only those beds $b \in \overline{B}$ that are scheduled to be vacated within the planning horizon *T*. This means that beds that are already occupied by patients who have an estimated LOS exceeding the planning horizon are not included ($\overline{B} \subseteq B$). Likewise, only those patients $p \in \overline{P}, \overline{P} \subseteq P$ who are not yet occupying a bed *b* within their designated ward space and who require a bed at some point in time within the planning horizon *T* are considered. In particular, this includes patients who have just arrived, patients who are already waiting in the overflow area, as well as future elective patients already scheduled and anticipated future emergency patients, at some point within the planning horizon *T*. Limiting the sets for patients and beds is possible, as non-medical room transfers are not allowed. Algorithm 1 demonstrates the pilot method tailored to the PBA problem.

**Algorithm 1:**
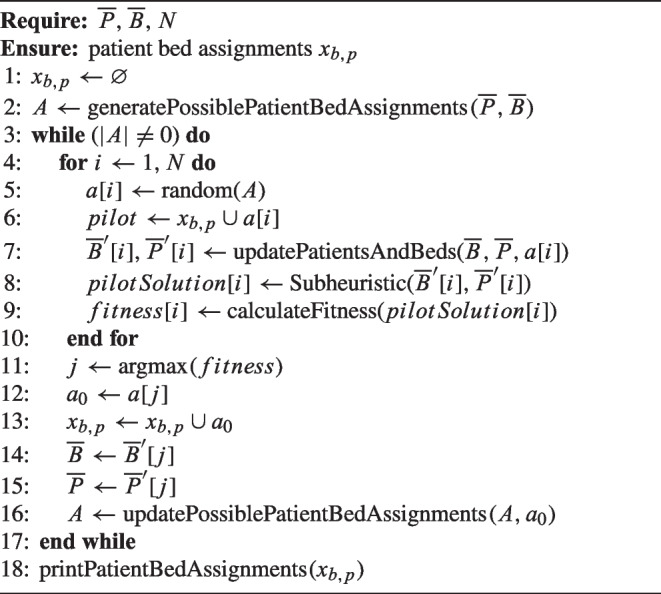
Pilot method for PBA.

#### Subheuristic

The subheuristic applied is based on the GLA heuristic developed by [40]. It sequentially calculates the potential added utility value with Eq. (1) of each possible patient bed combination and also considers at this stage the constraints in Eqs. (2) to (17). Finally, it executes the most promising assignment. The additional notation to describe the subheuristic is shown in Table 4.

**Table 4:** Further notation for the Subheuristic for PBA

$U_{b,p}$	Partial utility that an assignment of patient $p,p \in \overline{P}$ to bed $b,b\in \overline{B}$ may add to the total utility *U*
$U^{\textrm{argmax}}_p$	Index value of the bed *b* that adds the maximum partial utility $\max (U_{b,p})$ to the total utility *U* when patient *p*, $p \in \overline{P}$ is allocated to this bed *b*
$U^{\textrm{max}}_p$	Maximum partial utility that assignment of patient *p* adds to total utility *U*, $p \in \overline{P}$

**Table 5:** Overview of factors and properties assessed regarding correlation with emergency inpatient arrivals

Factor	Feature
Time and Date	Weekday ($\mathrm {WD_{Mon}}$, $\mathrm {WD_{Tue}}$, ...)
	Season (Q1, Q2, Q3, Q4)
	School holidays ($\mathrm {Hol_{School}}$)
	Bank holidays ($\textrm{Holiday}$)
	Post holiday weekday ($\mathrm {WD_{postholiday}})$
Weather	Temperature ($\mathrm {T_{mean}}$, $\mathrm {T_{min}}$, $\mathrm {T_{max}}$, $\mathrm {T_{dif}}$)
	Air pressure ($\mathrm {AP_{mean}}$, $\mathrm {AP_{min}}$, $\mathrm {AP_{max}}$, $\mathrm {AP_{dif}}$)
	Humidity ($\mathrm {H_{mean}}$, $\mathrm {H_{min}}$, $\mathrm {H_{max}}$, $\mathrm {H_{dif}}$)
	Wind ($\mathrm {W_{mean}}$, $\mathrm {W_{min}}$, $\mathrm {W_{max}}$, $\mathrm {W_{dif}}$, $\mathrm {G_{max}}$)
	Precipitation (Rain, Snow, Hail)
	Snow coverage ($\mathrm {S_{cov}}$)
	Storm
Local and Regional Events	Fairs (County Fairs, Sport events)
Current Occupancy	Admissions of previous day (PrevAdmin)

During an initialization process $x_{b,p}$ is set to zero and the utility matrix $U_{b,p}$ is calculated for all $p \in \overline{P}$ and $b \in \overline{B}$. The utility matrix $U_{b,p}$ represents partial utilities that can be added to the total utility function *U* (Objective function Eq. (1)) by realizing a patient *p* to bed *b* assignment. If a bed *b* is not available at any time of the planned stay for the specific patient *p*, the partial utility value $U_{b,p}$ is set to zero. In Iteration I (Step 1), the most promising combination $U_{b,p}$ is chosen, that yields the highest partial utility $U^{\textrm{max}}_p$, with $U^{\textrm{max}}_p = \textrm{max} \left( U_{b,p} \right) , \forall b \in \overline{B}, \forall p \in \overline{P}$. To accelerate the process of finding the highest value during the iterations, two auxiliary variables are used to indicate the uppermost potential utility of a patient’s assignment ($U^{\textrm{max}}_p$) and the corresponding bed ($U^{\textrm{argmax}}_p$). This reduces the amount of values that need to be compared from $|\overline{P}| \times |\overline{B}|$ to $|\overline{P}|$ in each step.

The initial allocation has an effect on a series of potential allocation combinations $x_{b,p}$ of the remaining patients *P* and beds *B*. Subsequently, in Iteration I (Step 2), potential patient bed utilities $U_{b,p}$ that have been affected by a previous PBA in Step 1 get updated. If necessary, $U^{\textrm{max}}_p$ and $U^{\textrm{argmax}}_p$ are redetermined. The following Iteration II (Step 1) also starts with the assignment of the most beneficial PBA. It will assign the patient first with the highest utility $U_{b,p}$. In Iteration II (Step 2), the utilities of all remaining patient bed combinations will be updated. This will be continued until all patients are assigned. Algorithm 2 represents the iterative, procedural program flow.

**Algorithm 2:**
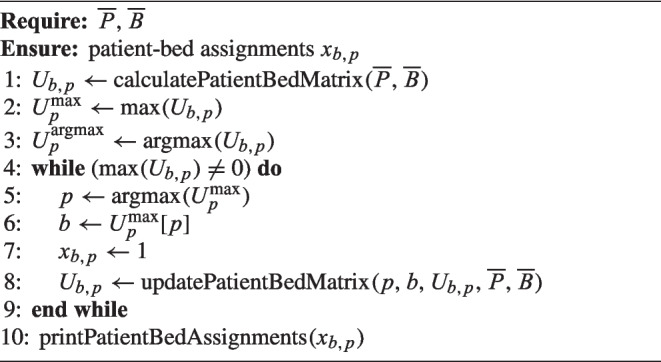
Subheuristic: GLA Heuristic for PBA.

#### Applied policies for patient bed assignment

To speed up the algorithm and tailor it to the PBA, different policies have been implemented and tested. First, at the start of each new pilot iteration the *filter policy* selects only a determined number of promising pilots. The vector $\textrm{argmax}(U_{p}^{\textrm{max}})$ (see Algorithm 2) is used for this, the calculation taking place anyway to subsequently complete the partial solutions. Here, only those pilots with high expected additional utility values are considered. Second, the *drop policy* is applied, which executes the subheuristic *H* for only a predetermined fraction of the remaining options $a \in A \setminus X$. This can be guaranteed by only considering patients in the subheuristic who arrive within a certain period (shorter than the planning horizon). Finally, we also restricted the *evaluation depth*, i.e., only a subset of pilots $a \subseteq A$ are allocated by the pilot method. The remaining ones $a \in A \setminus X$ get assigned by the subheuristic *H*. The efficiency and applicability of the different policies are investigated in the numerical studies.

## Numerical study

This section presents numerical studies. We draw upon real-life hospital data from a joint project with a large German hospital. First, we start in Subsection 4.1 by presenting the data and performing some basic tests. Second, we continue in Subsection 4.2 by presenting the TSF and ML approach used to anticipate emergency inpatient arrivals. Third, in Subsection 4.3 we show the performance of the hyper-heuristic we have developed. We compare it in different scenarios with the GLA developed by [40] as well as solutions obtained with Gurobi and a Genetic Algorithm (GA). Finally, in Subsection 4.4 we analyze the impact of both the enhanced emergency inpatient arrival forecasting approach as well as the improved hyper-heuristic on the overall solution. All computational steps were carried out using Gurobi 10.0, Python 3.10, and R 3.6. All computations were run on a work station equipped with an Intel Core i7-8550U processor and 16 GB of RAM.

### Overview of data

To analyze potential influences on emergency patient admissions, we have gathered metadata on various distinct features that were publicly available and which we suspected of having an impact on the emergency admissions. These features relate to time and dates, weather data, important local and regional events, as well as historical and current occupancy levels (see Table 5). We split the data set into training data which represents a time period of 2 years from 2014 to 2015, as well as test and validation data which is taken from 2016.

**Fig. 2:**
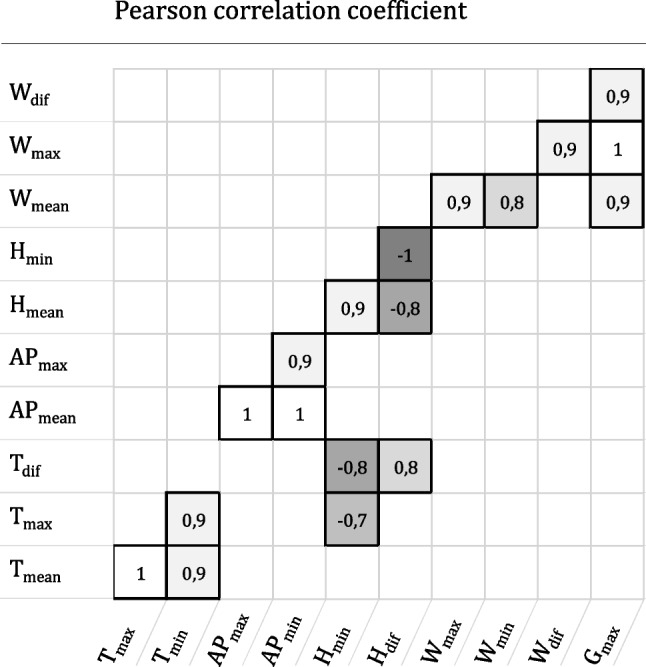
Measure of linear correlations between selected parameters

To determine the importance of the features we conduct the methodology as described in Section 3.1.1. The PCC is calculated for each potential pairing of features listed in Table 5. Figure 2 gives an overview of all problematic pairings, i.e., all pairings wherein $|\textrm{PCC}|>=0.7$. A simple example of this would be that the maximum temperature $\mathrm {T_{max}}$ strongly correlates with the minimum temperature $\mathrm {T_{min}}$, e.g., minimum and maximum temperatures for any given day during summer time are typically higher than during winter time. From now on one variable of the highly correlated pairings is neglected.

**Fig. 3:**
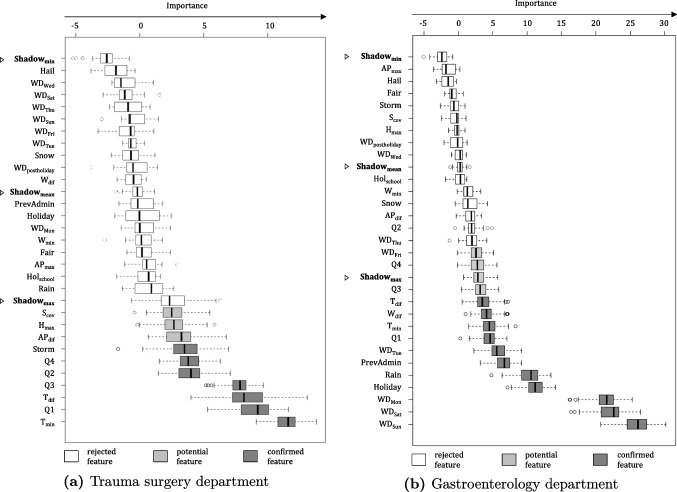
Trauma surgery department

As each medical department is expected to have its own drivers, the Boruta selection algorithm is individually executed for every medical department, that has emergency inpatient arrivals. To give an example, we present detailed results for two different departments, namely trauma surgery, and gastroenterology, as can be seen in Fig. 3a and b, respectively.

For trauma surgery, the number of emergency inpatient arrivals is clearly correlated with the seasons (Q1 to Q4), with low temperatures ($\mathrm {T_{min}}$), as well as with the magnitude of intra-day temperature changes ($\mathrm {T_{dif}}$). Naturally, any feature that correlates with the number of emergency inpatient arrivals, in both the training data set and the test data set, can prove useful when anticipating such arrivals. However, the causality behind this correlation may only be guessed. In the case of emergency patients having had an accident that requires trauma surgery, it seems plausible that sudden drops in temperature, which lead to black ice on roads and sidewalks, or typical recreational activities pursued in winter (Q1), e.g., skiing, are responsible for this effect.

For the gastroenterology department, however, the picture looks quite different. Here, holidays, weekends, and Mondays each exhibit a high explanatory correlation with regard to incoming emergency patients, whereas the temperature has a considerably lower influence when compared to the trauma surgery department. This could be due to a couple of different reasons. For instance, doctors and nursing staff we interviewed have reported that many gastroenterological illnesses often initially present with non-specific abdominal pain symptoms, which then intensify over several days. This means that in comparison with a broken hip, for example, there is no immediate need to get to a hospital, such that patients could opt to stay home on weekends. An alternative explanation could be that resident doctors’ offices are typically closed on weekends and patients who are not yet aware of the severity of their illness will usually wait until the next workday to see their family doctor who might then immediately refer them to a hospital for further treatment.

To summarize, the drivers for the arrival of emergency patients are different across departments. This requires to address the forecasting and PBA problem by department.

**Table 6:** Anticipation of emergency inpatient admissions using different forecasting methods

	Baseline	Approach 1 (TSF)		Approach 2 (ML)					Max.	
		Hol.Win.	SARIMA	RR	LR	EN	GL	ANN	Improvement	
Department	RMSE	RMSE	RMSE	RMSE	RMSE	RMSE	RMSE	RMSE	[%]	Type
Department 1	4.888	4.377	4.620	4.331	4.309	4.183	4.103	**4.060**	16.9	ANN
Department 2	4.108	3.963	4.087	3.892	3.847	3.848	**3.675**	3.835	10.5	GL
Department 3	2.888	2.920	2.811	2.887	2.824	2.800	**2.743**	2.778	5.0	GL
Department 4	3.535	3.222	3.677	3.126	3.099	3.097	**3.067**	3.192	13.2	GL

The bolded entries represent the best results obtained for each department using various methodologies

### Applying time series forecasting and ML to estimate emergency patients

We applied different forecasting approaches introduced in Section 3.1.2 to estimate emergency patient admissions. We first applied (1) TSF procedures. Second, to incorporate also the time series independent influencing factors described in Table 5 we deployed (2) regression-based methods. In addition, in a third step, we applied a (3) multilayer ANN to account for nonlinear dependencies. We used regularization methods in both approaches to avoid overfitting. Finally, we used the test data to evaluate the generalization abilities of our trained models.

#### (1) Time series forecasting

We started with TSF methods. Strong weekday dependencies made the use of methods that take seasonal effects into account indispensable. Therefore, we performed Holt-Winters’ triple exponential smoothing method [51] and applied a SARIMA model [10]. We used the procedure implemented by [31] to set the parameters for the SARIMA model. The selection of the best model is based on unit root tests and the Akaike Information Criterion (AIC).

#### (2) Regression-based methods

We used 10-fold cross-validation to tune the hyperparameter $\lambda$ for each approach, which controls the strength of the regularization. For EN we performed a grid-search between 0 and 1 in 0.025 steps to optimize the hyperparemters $\lambda _1$ and $\lambda _2$, which are used to control the $l_1$ and $l_2$ penalty respectively.

#### (3) Artificial neural network

We have evaluated several typologies of ANNs by varying the number of hidden layers between one and five. The best results have been achieved by applying a “32:16:8:4:2” network (the numbers are the number of neurons per hidden layer; hidden layers are separated by colons), the rectified linear unit (ReLu) as activation function, $l_1$ and $l_2$ regularization and the mean-squared error (MSE) loss function as well as the optimizer RMSprop. To avoid overfitting we have investigated the learning curve of training and validation loss. For tuning hyperparameters $l_1$ and $l_2$ we used a grid search algorithm.

#### Evaluation of performance on test data

We applied the models to the test data from four departments at our case hospital that have a significant number of emergency patients. For example, orthopedics has almost no emergency patients. We use the RMSE to compare the prediction accuracy of different models.

The historical averages serve as a baseline approach, and this is denoted as “Baseline”. This is compared with our above-described TSF (denoted as “Approach 1 (TSF)”) and ML approach (denoted as “Approach 2 (ML)”). Table 6 shows that the ML approach outperforms the baseline and “Approach 1”. The ML approach leads to improvements of up to 17%, depending on the department, compared to the basic historical averages.

The improvement obtained from the better forecasting can also be used as input in other bed-assignment problems like [40].

### Performance of the hyper-heuristic

In this section, we examine the performance of the developed hyper-heuristic by *(1) application to single problem instances*, *(2) application to rolling planning horizon*, and *(3) comparison versus optimal method and comparative heuristic*. In order to assess the solution quality of the hyper-heuristic proposed in this paper, we drew upon nine data sets. The available real data of one department cluster consisting of department 1 and department 2 (see also Section 4.2) based on actual patient movements between January 2016 and September 2016 will be considered. The cluster consists of five wards with 24 beds each. Each data set is composed of 28 consecutive days and comprises an average of 648 unique patients. On average, 40% of patients are men and 60% women with an average age of 70 years and a LOS of 6 days. All data sets reveal high ratios of emergency patients, e.g., up to 90%. The departments investigated are gastroenterology and internal medicine. Please note that we cannot show the actual data of our case hospital due to confidentiality reasons. We set the parameters in alignment with currently applied weights in our case hospital: $\alpha = 1$, $\beta = 0.1$, $\gamma , \delta = 2$, $Q_t=(1-q)^t$, $q = 0.01$, $|T|=7$. Furthermore, the weighting factor $\mathrm {\Xi }_p$ was set to three distinct values depending on the patient type. Notably, these consist of $\mathrm {\Xi }^{el} = 10$ for elective patients, $\mathrm {\Xi }^{em} = 9$ for current emergency admissions, and $\mathrm {\Xi }^{an} = 4$ for anticipated emergency inpatient arrivals. Here, elective patients are preferred to current emergency patients, and these in turn are given preference vs. expected future emergency admissions. We have adjusted the existing data by eliminating all uncertainty factors for the sole purpose of monitoring the performance of the heuristics applied. Accordingly, emergency patients are treated like elective patients and their exact admission is known in advance. Both patient types are no longer subject to LOS updates due to precisely known discharge times. Furthermore, patient no-shows are neglected. This means that the data sets considered are no longer affected by stochastic variations and are assumed to be deterministic.

#### (1) Application to single problem instances

We first assessed the performance of our hyper-heuristic for single problem instances. Using such a static version is a usual benchmark approach (see literature review above and for example in [9, 17, 23]). This approach excludes parameter-dependent (e.g., time-dependent relevance) performance differences caused by time series analysis. These parameters could lead to worse performance in the time series analysis and thus reduce the meaningfulness of hyper-heuristic performance despite better performance in all single problem instances. We tested several policies (see Section 3.3). In particular, we applied a *filter policy* with which we restricted the number of promising pilots to different predetermined amounts, which were determined based on their individual additional potential benefit to the utility function before an algorithm run-through. The best patient-bed assignments are drawn randomly from the five most promising patients. This was done to avoid unnecessary computational effort while at the same time ensuring that no potentially “lucrative” PBAs are overlooked. It should be noted that several potential PBAs of a single patient may have similar values and hence a wide variety of alternative promising PBAs exist. In addition, we applied a *drop policy* by limiting the application of the GLA subheuristic to only those patients that were known or anticipated to arrive within a certain number of days, which also leads to a significant reduction of computational time while retaining a high solution quality. Finally, we varied the *evaluation depth* by restricting the number of subsequent PBAs obtained through the pilot method. To give an example, selecting only 10 pilots and a depth of 20 translates into applying the pilot method to determine the first 20 PBA, wherein for each of these 20 assignments the 10 most promising pilots will be chosen and evaluated using the GLA heuristic.

Table 7 gives an overview of the solutions obtained. For each of the shown combinations of data set used, amount of promising pilots filtered (in lines), and evaluation depth (in columns), we have taken into account all single problem instances which emerged by executing the data sets. This results in around 2,000 single problem instances for each data set (i.e., around 288,000 in total), promising pilot and evaluation depth combinations. We did this to account for statistical distributions, which arise due to the inherent randomness associated with our implementation of the hyper-heuristic.

**Table 7:** Solution quality of the Pilot method compared to the GLA heuristic for single problem instances

DS$^{1}$ 1	Depth					DS 2	Depth				
Pilots $^{2}$	5	10	15	20	Avg.	Pilots	5	10	15	20	Avg.
5	1.41%	1.96%	2.12%	2.13%	1.91%	5	1.30%	1.78%	2.04%	2.23%	1.84%
10	1.60%	1.79%	2.29%	2.59%	2.07%	10	1.27%	1.87%	2.06%	2.29%	1.87%
15	1.67%	2.06%	2.11%	2.89%	2.18%	15	1.36%	2.25%	2.38%	2.54%	2.13%
20	2.19%	2.20%	2.74%	2.88%	2.50%	20	1.87%	2.48%	2.60%	2.59%	2.39%
Avrg	1.72%	2.00%	2.32%	2.62%	-	Avrg	1.45%	2.10%	2.27%	2.41%	-
DS 3	Depth					DS 4	Depth				
Pilots	5	10	15	20	Avg.	Pilots	5	10	15	20	Avg.
5	1.53%	1.77%	1.91%	1.93%	1.79%	5	1.25%	1.93%	2.06%	2.02%	1.82%
10	1.37%	1.72%	1.86%	2.82%	1.94%	10	1.25%	2.08%	2.29%	2.50%	2.03%
15	1.26%	2.33%	2.75%	2.97%	2.33%	15	1.50%	2.39%	2.59%	3.14%	2.41%
20	1.80%	2.52%	2.79%	3.13%	2.56%	20	1.91%	2.40%	2.46%	3.26%	2.51%
Average	1.49%	2.09%	2.33%	2.71%	-	Average	1.48%	2.20%	2.35%	2.73%	-
DS 5	Depth					DS 6	Depth				
Pilots	5	10	15	20	Avg.	Pilots	5	10	15	20	Avg.
5	1.13%	1.50%	1.80%	2.04%	1.62%	5	1.63%	1.91%	1.98%	1.99%	1.88%
10	1.28%	1.60%	1.87%	2.05%	1.70%	10	1.51%	2.04%	2.07%	2.06%	1.92%
15	1.10%	1.46%	2.07%	2.29%	1.73%	15	1.84%	1.97%	2.09%	2.17%	2.02%
20	1.64%	2.18%	2.01%	2.39%	2.05%	20	2.11%	2.04%	2.03%	2.25%	2.11%
Average	1.29%	1.68%	1.94%	2.19%	-	Average	1.77%	1.99%	2.04%	2.12%	-
DS 7	Depth					DS 8	Depth				
Pilots	5	10	15	20	Avg.	Pilots	5	10	15	20	Avg.
5	1.30%	1.79%	1.98%	2.28%	1.84%	5	1.29%	1.94%	2.22%	2.26%	1.93%
10	1.42%	2.22%	2.04%	2.32%	2.00%	10	1.42%	2.11%	2.22%	2.33%	2.02%
15	1.56%	2.08%	2.63%	2.76%	2.26%	15	1.43%	2.30%	2.46%	2.37%	2.14%
20	2.22%	2.69%	2.71%	3.30%	2.73%	20	1.84%	2.44%	3.20%	3.57%	2.76%
Average	1.62%	2.19%	2.34%	2.67%	-	Average	1.49%	2.20%	2.53%	2.63%	-
DS 9	Depth					Total$^{3}$	Depth				
Pilots	5	10	15	20	Avg.	Pilots	5	10	15	20	Avg.
5	1.20%	1.58%	1.94%	2.00%	1.68%	5	1.34%	1.80%	2.00%	2.10%	1.81%
10	1.19%	1.67%	1.88%	2.06%	1.70%	10	1.37%	1.90%	2.07%	2.34%	1.92%
15	1.41%	1.77%	2.15%	2.49%	1.96%	15	1.46%	2.07%	2.36%	2.62%	2.13%
20	1.94%	2.44%	2.48%	2.70%	2.39%	20	1.94%	2.38%	2.56%	2.90%	2.44%
Average	1.43%	1.87%	2.11%	2.31%	-	Average	1.53%	2.04%	2.25%	2.49%	-

Calculation: Hyper-heuristic utility / GLA utility - 1

$^{1}$ Data set used to extract problem instances

$^{2}$ Number of promising pilots filtered for further analysis

$^{3}$ Total average across all problem instances analyzed

**Table 8:** Solution quality of Pilot method compared to GLA heuristic for rolling horizon

Data	24 beds			Data	48 beds		
Set	Min	Avg.	Max	Set	Min	Avg.	Max
1	0.88%	2.23%	4.68%	1	2.17%	3.48%	5.04%
2	0.66%	1.16%	2.03%	2	-0.03%	2.22%	3.59%
3	-0.94%	0.11%	0.99%	3	0.95%	1.30%	1.77%
4	1.73%	2.11%	2.47%	4	2.43%	4.13%	4.83%
5	0.18%	1.42%	3.49%	5	1.84%	3.00%	3.70%
6	2.21%	3.30%	4.07%	6	2.31%	2.64%	3.14%
7	2.45%	2.61%	2.88%	7	2.53%	3.51%	5.30%
8	1.32%	2.78%	3.92%	8	1.64%	3.97%	5.10%
9	-0.59%	0.61%	1.98%	9	1.65%	2.79%	3.92%
Avg.	0.88%	1.81%	2.95%	Avg.	1.72%	3.00%	4.04%
Data	72 beds			Data	96 beds		
Set	Min	Avg.	Max	Set	Min	Avg.	Max
1	1.08%	2.20%	3.26%	1	1.36%	2.30%	2.95%
2	1.81%	3.16%	4.36%	2	1.39%	2.21%	2.67%
3	2.07%	2.48%	2.84%	3	-0.01%	0.34%	0.68%
4	1.18%	1.66%	2.14%	4	1.15%	1.64%	2.06%
5	2.54%	2.61%	2.72%	5	2.04%	2.47%	2.74%
6	1.16%	1.81%	2.23%	6	1.22%	2.58%	3.65%
7	1.77%	2.78%	3.36%	7	2.29%	2.57%	2.95%
8	2.25%	2.65%	3.07%	8	0.48%	1.24%	2.04%
9	1.25%	1.36%	1.43%	9	0.26%	0.85%	1.13%
Avg.	1.68%	2.30%	2.82%	Avg.	1.13%	1.80%	2.32%
Data	120 beds			Data	Total$^{1}$		
Set	Min	Avg.	Max	Set	Min	Avg.	Max
1	1.79%	2.40%	2.90%	1	1.46%	2.52%	3.77%
2	1.28%	1.80%	2.28%	2	1.02%	2.11%	2.99%
3	1.52%	2.35%	3.02%	3	0.72%	1.32%	1.86%
4	-0.19%	0.79%	1.45%	4	1.26%	2.07%	2.59%
5	1.02%	1.35%	1.76%	5	1.52%	2.17%	2.88%
6	2.83%	3.38%	4.03%	6	1.95%	2.74%	3.42%
7	1.78%	2.22%	2.80%	7	2.16%	2.74%	3.46%
8	-0.07%	0.37%	0.79%	8	1.12%	2.20%	2.99%
9	1.11%	1.56%	2.06%	9	0.74%	1.43%	2.10%
Avg.	1.23%	1.80%	2.34%	Avg.	1.33%	2.14%	2.90%

Calculation: Hyper-heuristic utility / GLA utility - 1

$^{1}$Total average across all bed sizes

The results obtained (see Table 7) allow for drawing three main insights. First, by using the pilot method, it was possible to increase the solution quality in comparison to the GLA heuristic by up to 3.57% while achieving an average increase of 2.90% when considering 20 promising pilots combined with an evaluation depth of 20. This number can of course vary depending on the characteristics of the underlying patient clientele. However, the effect observed is substantially the same across all nine data sets. Second, as is to be expected, increasing the evaluation depth as well as increasing the number of promising pilots both lead to an increase in solution quality. This is because it is more likely that better solutions will be found when broadening the search space as this increases the chance of finding solutions that are further away from standard GLA heuristic solutions. The effect of increasing the evaluation depth has a higher impact on solution quality than increasing the number of promising pilots considered. A reason for this effect could be seen in that even when using a low number of promising pilots considered, the pilots chosen exhibit the highest additional benefit to the overall utility function, respectively, which makes the underlying PBA more likely to be part of a good solution. Third, depending on the situation at hand, the acquired gain in solution quality due to a broader search space goes hand in hand with higher computational effort, which can be an important factor when requiring real-time PBAs in actual applications. Roughly speaking, the total computation time for a single problem instance can be estimated by adding up the total number of times the subheuristic has to run through all PBAs for a given single problem instance. To give an example, an evaluation depth of 10 combined with 10 selected pilots will add up to 100 applications of the subheuristic while an evaluation depth of 5 combined with 5 selected pilots will only require 25 run-throughs of the GLA heuristic, or 25% of the time. The run time changes are only proportional to the dimension of evaluation depth when multi-processing is applied. This means that the run time compared to the GLA heuristic is just multiplied by the evaluation depth. The GLA heuristic is typically solved in an average of less than one second for instances encompassing 120 beds.

**Table 9:** Solution quality of the Hyper-Heuristic compared to benchmarks using single problem instances

	Number of beds											
	solution quality$^{1}$ [%] | run time$^{2}$ [sec]											
Approach	24		48		72		96		120		Total $^{3}$	
GLA	100.00	0.03	100.00	0.12	100.00	0.24	100.00	0.37	100.00	0.60	100.00	0.27
GA	103.86	114.64	103.57	300.00	101.88	300.00	101.26	300.00	101.67	300.00	102.45	262.93
Hyper-Heuristic	104.15	24.13	103.66	106.74	103.57	186.66	102.98	223.79	103.16	279.02	103.50	164.07
Gurobi	105.99	300.00	110.47	300.00	105.19	300.00	NFS $^{4}$	300.00	NFS $^{4}$	300.00	(107.22) $^{5}$	300.00

$^{1}$ Calculated as total average of utility obtained with solution approach / utility obtained with GLA

$^{2}$ Run time limited to 300 seconds; 300 indicates time limit violation

$^{3}$ Total average across all number of beds

$^{4}$ NFS := no feasible solution found

$^{5}$ Average across 24, 48 and 72 beds

#### (2) Application to rolling planning horizon

In addition to comparing the solution quality for single problem instances, we have undertaken analyses to compare the performances of both approaches over time. For this purpose, the data sets that have been cleared of uncertainties are also used. Furthermore, to investigate the scaling effect in relation to the department cluster size we divided the nine existing data sets with regard to the department cluster size stepwise by 24 beds from 24 to 120. To do this, the patients and beds are added depending on the division of the wards and their specific specialty. To test the hyper-heuristic approach developed, we use the top-performing settings from the single problem instance analyses (see Table 7), i.e., an evaluation depth of 20 combined with a selection of 20 promising pilots for each subsequent PBA.

The results of this analysis are presented in Table 8. Again, we have accounted for the statistical effects of the stochastic search procedure by running the algorithm 20 times for each combination of data set and beds considered. Here, the results show an increase in total utility. The hyper-heuristic approach outperforms the GLA heuristic by 2.14% on average while achieving an increase of up to 5.30% for certain data sets. The utility increase of the hyper-heuristic vs. the GLA heuristic for the time series analyses in Table 8 is not as clearly predictable as for the single problem instance solution in Table 7. This is due to the settings of the hyper-heuristic (i.e., planning horizon, time-dependent relevance parameter $Q_t$). Furthermore, only patients within the planning horizon, that may overlap with the hospital stays of future elective patients (arrival exceeding the planning horizon) are considered. In other words, even if the hyper-heuristic performs considerably better than the GLA heuristic for each single problem instance within the time series investigated, time-dependent parameter settings may eradicate the positive effect of the hyper-heuristic compared to the GLA heuristic for certain combinations of data sets and beds. This also explains some negative entries in the minimum values of Table 8. The hyper-heuristic outperformed the GLA heuristic in over 99% of the test instances.

**Table 10:** Solution quality of the Hyper-Heuristic ML compared to benchmarks using a rolling horizon

Model	Data Set				
	1	2	3	4	5
GLA Avg	105.81%	102.63%	102.26%	111.13%	97.97%
GLA ML	106.90%	102.76%	103.60%	111.17%	100.83%
Hyper-Heuristic ML	107.28%	103.53%	104.51%	111.78%	101.06%
	Data Set				
	6	7	8	9	Total$^{1}$
GLA Avg.	97.26%	98.17%	90.84%	93.92%	100.00%
GLA ML	100.28%	99.41%	92.62%	95.53%	101.45%
Hyper-Heuristic ML	100.39%	99.77%	93.86%	96.11%	102.30%

Calculation: Data Set utility / GLA utility Total Avg

$^{1}$Total average across all data sets

#### (3) Comparison of hyper-heuristic versus optimal method and comparative heuristic

This section further analyzes the run time and solution efficiency of the hyper-heuristic proposed versus the optimal solution and a comparative metaheuristic. For the comparison with the optimal solution, we implemented the model in Gurobi. As an additional comparison heuristic we implemented a Genetic Algorithm (GA). The GA has also been applied successfully in the most recent PBA literature and hence constitutes a benchmark approach form the literature. Dorgham et al. [17] and Alfred and Yu [2] show the efficiency of the GA in the PBA context. The parameters for the GA are set for the population size $= 50$, number of generations $= 100$, crossover probability $= 0.8$, and mutation probability $= 0.2$. Furthermore, we adopt the principle of elitism. The patient occupations resulting from the GLA method are inserted into the population as individuals in the first generation of the GA. Therefore, the results of the GA are always at least as good as those of the GLA. To be suitable for the PBA problem, repair mechanisms are built into all GA operators.

The comparison considers 10 individual instances drawn randomly from the nine data sets for each of the 5 scenarios ranging from 24 to 120 beds. It is ensured that the initial situation is the same for comparison methods. For this purpose, prior decisions are made using the GLA heuristic before applying one of the corresponding comparison methods. Since the hyper-heuristics and the GA are based on stochastic elements, every single instance is solved 20 times for these methods. All methods are limited to a maximum run time of 300 seconds. The best feasible solution found to this point is used for the comparison.

Table 9 outlines the results. Gurobi was unable to prove the optimality of its results in any of the sample instances. The mixed-integer programming (MIP) gap was on average 4%, 16%, and 29% for the instances with 24, 48, and 72 beds, respectively. Nevertheless, Gurobi found higher objective values for these small problems. For the instances with more beds, Gurobi could not find a feasible solution within the time limit. The hyper-heuristic outperforms the GA in both run time and solution quality. The run time is more than five times lower for instances where the run time limit is not reached by the GA.

### Hyper-heuristic combined with enhanced emergency inpatient arrival forecasting

In this subsection, the impact of both the enhanced emergency inpatient arrival forecasting approach as well as the improved hyper-heuristic with regard to real data are analyzed. The nine data sets (5 wards with 24 beds each), including uncertainties, are used to do this. Each data set consists of around 1,500 unique events that take place over 21 days. The LOS of the estimated emergency admissions is set to the median emergency LOS of the specialty. We denote the hyper-heuristic approach including the enhanced emergency patient admission data, which was achieved with ML, as *Hyper-Heuristic ML*. It is executed 20 times for all data sets and the average of all runs is reported. We will not include the GA in this section because the hyper-heuristic outperforms it in both solution quality and runtime. Also Gurobi is not a viable option because it is unable to find a feasible solution within a reasonable amount of time, especially for large problem sizes and computation time limits for an operational planning problem. Instead, we will use the GLA as a comparison, as it is the most efficient algorithm in terms of runtime. We apply two benchmarks:

- GLA Avg: The first is the GLA heuristic of [40] where the admissions of emergency patients have been estimated according to Approach 1 (see Section 4.2). This is exactly the approach in [40]. We normalize all values of the alternative approaches to this.
- GLA ML: The second is also based on the GLA heuristic, but the admissions of emergency patients have also been estimated with ML.

Looking at the results of the analysis of the three methods in Table 10, the normalized values of the objective function give a first indication of the performance of our approach. It can be noted that the Hyper-Heuristic ML outperforms the GLA as well as the GLA ML approach in each data set. On average across all data sets the Hyper-Heuristic ML shows 1.4% better results than the GLA and beats the GLA ML by 0.4%. Even the minimum outcome of the Hyper-Heuristic ML for all data sets performs better than the GLA method. This makes the Hyper-Heuristic ML the most promising and reliable approach to solving the PBA problem.

## Conclusion and further areas of research

### Conclusion

ML is revolutionizing healthcare management due to the exponential increase of data and computing power [3]. Predictive analytics will be entering the space of operational management in hospitals. In this chapter we highlight the impact on PBA. This paper develops and investigates improvements for the operational PBA. The model used has been developed in a joint project with a large German hospital covering all major disciplines and incorporates the objectives and constraints of the three main stakeholders, namely patients, doctors, and nursing staff. It integrates the planning of current emergency and elective patient admission, future elective patient admission, as well as anticipated future emergency patient admission. Three important aspects were tackled and improved in this paper.

- To tackle the uncertainty of emergency patient admissions, we applied ML techniques to estimate these more precisely. To this end, we used historic emergency inpatient data as well as metadata relating to time, date, weather forecasts, and local and regional events. We are the first to investigate and make use of the correlation of several external factors, such as weather data, to better anticipate emergency inpatient admissions.
- To enhance the performance of the solution approach, we tailor a hyper-heuristic to our problem setting.
- To assess the impact of advanced forecasts, we combine ML for forecasting emergency patient admissions with advanced optimization through the use of a hyper-heuristic approach, and its deployment in real-world applications.

Our numerical results have shown that ML approaches can outperform historical average approaches by up to 17% when it comes to predicting emergency inpatient arrivals (see Table 6). The underlying drivers for emergency inpatient arrivals differ strongly between departments due to the associated patient clientele, e.g., Trauma Surgery shows a higher dependency on weather data than Gastroenterology, which in turn is more strongly correlated with times and dates. Compared to the GLA heuristic, the hyper-heuristic developed can improve performance by up to 3.6% for single problem instances and up to 5.3% in a time series analysis. With respect to real data, the hyper-heuristic approach combined with a sophisticated prediction of future emergency patient admissions by ML outperforms the GLA heuristic in a time series analysis by up to 3.3%.The improvement obtained from the better forecasting can also be used as input in other bed-assignment problems.

### Future areas of research

Various opportunities exist for further research. For the problem shown, the existing solution methods can be further developed and different approaches can be pursued. The focus may be on enhanced anticipation of the input parameters, improvement of the heuristic methods, or development of an optimal solution method. The estimate of input parameters focuses on both emergency and elective patients. Information on the progress the patient’s recovery is making (e.g., LOS as well as type and probability of complications) can be anticipated for both patient groups. The approximation of time-related arrivals and patient characteristics (e.g., gender, age, and disease) is especially in focus for emergency patients, while no-show rates are interesting for elective patients. Another topic of research interest is to integrate upstream and/or downstream processes in the decision model (see e.g., [29], such as patient admission scheduling (see e.g., [30]), operating room scheduling (see e.g., [4, 21, 38]), bed transport services (see e.g., [7, 42]) or staff rostering (see e.g., [19, 45]). This integration makes it possible to obtain information about conflicts of interests of individual problems. In order to maximize profit, operating rooms should usually be booked to full capacity, although the hospital may not have suitable beds available for patients who have had surgery. Furthermore, the underlying mechanics of the PBA decision model are not limited to hospital settings alone. Further investigation could be made into identifying problem settings that have a similar scope. To give an example, the student-room assignment problem in hostels [2] could potentially yield further areas of application.
